# Supplementary material for: The Systems Biology Research Tool: evolvable open-source software
Source: BMC Syst Biol. 2008 Jun 29;2:55. doi: 10.1186/1752-0509-2-55 (PMC2446383; doi:10.1186/1752-0509-2-55)
Supplement: Additional file 1 — SBRT Archive. An archive of the current version of the Systems Biology Research Tool. [file 1752-0509-2-55-S1.zip › sbrt-1.4.0/doc/developers_guide/api/sbrt/kernel/fba/comp/RxnDeletionComputer.html]

RxnDeletionComputer


|  |  |  |
| --- | --- | --- |
| **Overview** \| **Package** \| **Class** \| **Use** \| **Tree** \| **Deprecated** \| **Index** \| **Help** | |  |
| **PREV CLASS**   **NEXT CLASS** | **FRAMES**    **NO FRAMES**     **All Classes** |  |
| SUMMARY: NESTED \| FIELD \| CONSTR \| METHOD | DETAIL: FIELD \| CONSTR \| METHOD |  |


---


## sbrt.kernel.fba.comp Class RxnDeletionComputer

```
java.lang.Object
  sbrt.kernel.fba.comp.AbstractCvComputer
      sbrt.kernel.fba.comp.RxnDeletionComputer
```

**All Implemented Interfaces:**: FluxCvComputer

---

``` public class RxnDeletionComputer extends AbstractCvComputer ```

This class is used to compute the effect of deleting reactions in a
fluxome.

**Author:**
:   This class was written and documented by Jeremiah Wright
    while in the Wagner lab.

---

**Constructor Summary**

`RxnDeletionComputer(FbaOptComputer computer)`
  
          Constructs a new reaction deletion computer.


| **Method Summary** | |
| --- | --- |
| `FbaOptComputer.Results` | `delete(java.util.Set<java.lang.String> rxnNames)`             Computes and returns the results of deleting the reactions whose names appear in the provided set. |
| `Interval` | `getFluxConstraint(java.lang.String rxnName)`             Returns the flux constraint of the reaction with the provided name. |
| `java.util.Map<java.lang.String,Interval>` | `getFluxConstraints()`             Returns the set of all constraints on single fluxes. |
| `MathExpr<java.lang.String>` | `getObjectiveFunction()`             Returns the current objective function. |
| `OptimizationSense` | `getOptimizationSense()`             Returns the current optimization sense. |
| `Interval` | `getRxnExprConstraint(MathExpr<java.lang.String> rxnNameExp)`             Returns the constraint on the provided mathematical expression of fluxes. |
| `java.util.Map<MathExpr<java.lang.String>,Interval>` | `getRxnExprConstraints()`             Returns the set of all constraints on mathematical expressions of fluxes. |
| `double` | `getZeroCutoff()`             Returns the current zero cutoff. |
| `void` | `restoreFluxConstraint(java.lang.String reactionName)`             Restores the flux constraint for the reaction with the provided name to the value contained in the fluxome. |
| `void` | `restoreRxnExprConstraint(MathExpr<java.lang.String> rxnNameExp)`             Restores the constraint on the provided flux expression to the value contained in the fluxome. |
| `void` | `restoreRxnExprConstraints()`             Restores the constraints on all flux expressions to the values contained in the fluxome. |
| `void` | `setFluxConstraint(java.lang.String reactionName, Interval c)`             Changes the flux constraint of the reaction with the provided name. |
| `void` | `setObjectiveFunction(MathExpr<java.lang.String> objective)`             Sets the objective function to the provided mathematical expression. |
| `void` | `setOptimizationSense(OptimizationSense sense)`             Sets the optimization sense to the provided value. |
| `void` | `setRxnExprConstraint(MathExpr<java.lang.String> e, Interval c)`             Changes the constraint on the provided mathematical expression of fluxes. |
| `void` | `setZeroCutoff(double zeroCutoff)`             Sets the zero cutoff to the provided value. |

**Methods inherited from class sbrt.kernel.fba.comp.AbstractCvComputer**

`checkFluxConstraint, checkNumber, checkRxnExpr, checkRxnExprConstraint, checkRxnName, checkRxnNames, getFluxome, getRxnExpr, getRxnExpr, restoreFluxConstraints, restoreFluxConstraints, restoreRxnExprConstraints, setFluxConstraints, setRxnExprConstraints`

**Methods inherited from class java.lang.Object**

`clone, equals, finalize, getClass, hashCode, notify, notifyAll, toString, wait, wait, wait`

**Constructor Detail**

### RxnDeletionComputer

```
public RxnDeletionComputer(FbaOptComputer computer)
```

:   Constructs a new reaction deletion computer.

    **Parameters:**: `computer` - the FBA optimization computer to use for all optimizations.


**Method Detail**

### setZeroCutoff

```
public void setZeroCutoff(double zeroCutoff)
```

:   Sets the zero cutoff to the provided value. The zero cutoff is the
    value used to decide which computed values are close enough to zero
    to be considered equal to zero. This value is an upper bound and
    should itself be close to zero, like 1E-6 for example.

    :   **Parameters:**: `zeroCutoff` - the zero cutoff.

---


### setOptimizationSense

```
public void setOptimizationSense(OptimizationSense sense)
```

:   Sets the optimization sense to the provided value.

    :   **Parameters:**: `sense` - an optimization sense.

---


### setObjectiveFunction

```
public void setObjectiveFunction(MathExpr<java.lang.String> objective)
```

:   Sets the objective function to the provided mathematical expression.

    :   **Parameters:**: `objective` - a mathematical expression of reaction names.

---


### getObjectiveFunction

```
public MathExpr<java.lang.String> getObjectiveFunction()
```

:   Returns the current objective function.

    :   **Returns:**: the current objective function.

---


### getOptimizationSense

```
public OptimizationSense getOptimizationSense()
```

:   Returns the current optimization sense.

    :   **Returns:**: the current optimization sense.

---


### getZeroCutoff

```
public double getZeroCutoff()
```

:   Returns the current zero cutoff.

    :   **Returns:**: the current zero cutoff. **See Also:**: `setZeroCutoff(double)`

---


### delete

```
public FbaOptComputer.Results delete(java.util.Set<java.lang.String> rxnNames)
```

:   Computes and returns the results of deleting the reactions whose names
    appear in the provided set.

    :   **Parameters:**: `rxnNames` - the names of reactions whose effect upon deletion will be computed. **Returns:**: the results of deleting the reactions whose names occur in the provided set. **Throws:**: `java.lang.IllegalStateException` - if no objective function or optimization sense were provided.

---


### getRxnExprConstraints

```
public java.util.Map<MathExpr<java.lang.String>,Interval> getRxnExprConstraints()
```

:   Returns the set of all constraints on mathematical expressions of
    fluxes.

    :   **Returns:**: mathematical expressions of reaction names mapped to their corresponding constaint.

---


### getFluxConstraints

```
public java.util.Map<java.lang.String,Interval> getFluxConstraints()
```

:   Returns the set of all constraints on single fluxes.

    :   **Returns:**: the names of reaction in the fluxome mapped to their corresponding flux constaint.

---


### getRxnExprConstraint

```
public Interval getRxnExprConstraint(MathExpr<java.lang.String> rxnNameExp)
```

:   Returns the constraint on the provided mathematical expression of
    fluxes.

    :   **Parameters:**: `rxnNameExp` - a mathematical expression of reaction names. **Returns:**: the constraint on the provided mathematical expression of fluxes.

---


### getFluxConstraint

```
public Interval getFluxConstraint(java.lang.String rxnName)
```

:   Returns the flux constraint of the reaction with the provided name.

    :   **Parameters:**: `rxnName` - the name of the reaction whose flux constraint is to be returned. **Returns:**: the flux constaint of the reaction with the provided name.

---


### setFluxConstraint

```
public void setFluxConstraint(java.lang.String reactionName,
                              Interval c)
```

:   Changes the flux constraint of the reaction with the provided name.

    :   **Parameters:**: `reactionName` - the name of the reaction whose flux constraint is to be changed.: `c` - the new constraint.

---


### setRxnExprConstraint

```
public void setRxnExprConstraint(MathExpr<java.lang.String> e,
                                 Interval c)
```

:   Changes the constraint on the provided mathematical expression
    of fluxes.

    :   **Parameters:**: `e` - a matheamtical expression of reaction names.: `c` - the new constraint.

---


### restoreRxnExprConstraints

```
public void restoreRxnExprConstraints()
```

:   Restores the constraints on all flux expressions to the values
    contained in the fluxome.

---


### restoreFluxConstraint

```
public void restoreFluxConstraint(java.lang.String reactionName)
```

:   Restores the flux constraint for the reaction with the provided
    name to the value contained in the fluxome.

    :   **Parameters:**: `reactionName` - the name of the reaction whose flux constraint is to be restored.

---


### restoreRxnExprConstraint

```
public void restoreRxnExprConstraint(MathExpr<java.lang.String> rxnNameExp)
```

:   Restores the constraint on the provided flux expression to the value
    contained in the fluxome.

    :   **Parameters:**: `rxnNameExp` - the expression of reaction names whose contraint is to be restored.


---


|  |  |  |
| --- | --- | --- |
| **Overview** \| **Package** \| **Class** \| **Use** \| **Tree** \| **Deprecated** \| **Index** \| **Help** | |  |
| **PREV CLASS**   **NEXT CLASS** | **FRAMES**    **NO FRAMES**     **All Classes** |  |
| SUMMARY: NESTED \| FIELD \| CONSTR \| METHOD | DETAIL: FIELD \| CONSTR \| METHOD |  |


---
